# Oral antibiotics with early hospital discharge compared with in-patient intravenous antibiotics for low-risk febrile neutropenia in patients with cancer: a prospective randomised controlled single centre study

**DOI:** 10.1038/sj.bjc.6600993

**Published:** 2003-07-01

**Authors:** H E Innes, D B Smith, S M O'Reilly, P I Clark, V Kelly, E Marshall

**Affiliations:** 1 Clatterbridge Centre for Oncology, Bebington, Wirral, Merseyside, UK

**Keywords:** low-risk, febrile neutropenia, oral antibiotics, outpatient

## Abstract

Neutropenic sepsis remains a potentially life-threatening complication of anticancer chemotherapy. However, it is possible to identify patients who are at low risk for serious complications and for whom less-intensive, more-convenient treatment may be appropriate. The aim of this study was to assess the efficacy and safety of oral antibiotics in conjunction with early hospital discharge in comparison with standard in-patient intravenous antibiotics in patients with low-risk neutropenic fever. In all, 126 episodes of low-risk neutropenic fever occurred in 102 patients. Patients were randomised to receive either: an oral regimen of ciprofloxacin (750 mg 12 hourly) plus amoxicillin–clavulanate (675 mg 8 hourly) for a total of 5 days, or a standard intravenous regimen of gentamicin and tazocin (piperacillin/tazobactam) until hospital discharge. Patients randomised to oral antibiotics were eligible for discharge following 24 h of hospitalisation, if clinically stable and symptomatically improved. The efficacy of the two arms was similar: initial treatment was successful without antibiotic modification in 90% of episodes in the intravenous arm and 84.8% of episodes in the oral arm, *P*=0.55, absolute difference between the groups 5.2%; 95% confidence interval (CI) for the difference −7 to 17.3%. Only one episode in the oral arm was associated with significant clinical deterioration: this occurred within the initial in-patient assessment period. The median in-patient stay was 4 days in the intravenous arm (range 2–8) and 2 days in the oral arm (range 1–16 days), *P*<0.0005. The reduction in hospital stay led to significant cost-savings in the oral arm. In conclusion, this study suggests that oral antibiotics in conjunction with early hospital discharge for patients who remain stable after a 24 h period of in-patient monitoring offers a feasible and cost-effective alternative to conventional management of low-risk neutropenic fever.

Neutropenic sepsis is a well recognised, potentially life-threatening complication for patients undergoing cytotoxic chemotherapy for cancer (Bodey *et al*, 1966). As a consequence, prompt in-patient therapy with broad-spectrum intravenous antibiotics has become the standard of care for all patients who develop febrile episodes while neutropenic (Hughes *et al*, 1990, 1997). While this approach has undoubtedly reduced mortality from sepsis, it has become increasingly recognised that febrile neutropenia represents a spectrum of potential severity and that only a relatively small proportion of patients are at high risk of complications or death, the great majority of febrile episodes running a benign course. Several groups of investigators have independently developed prognostic indices in neutropenic fever, in an attempt to identify criteria by which to define ‘low-risk’ (Rubin *et al*, 1988; Talcott *et al*, 1988, 1992; Viscoli *et al*, 1994; Elting *et al*, 1997). More recently, these groups have come together in an international collaboration with the publication of a ‘risk index’ (Klastersky *et al*, 2000) using a weighted scoring system of clinical factors, based upon analysis of 1139 episodes of neutropenia.

The aim of defining low-risk neutropenia has been to identify those patients who may be candidates for less-intensive, more-convenient antibiotic therapy. Thus, in parallel with the development of the definition of low-risk, several studies have demonstrated the feasibility of newer approaches including intravenous antibiotic monotherapy (Pizzo *et al*, 1986; Sanders *et al*, 1991; Rolston *et al*, 1992; Marshall *et al*, 2000), outpatient ambulatory intravenous therapy (Rubenstein *et al*, 1993; Talcott *et al*, 1994) and the use of oral antibiotic regimens (Gardembas-Pain *et al*, 1991; Malik *et al*, 1992). These approaches have recently culminated in the publication of two large prospective randomised trials (Freifeld *et al*, 1999; Kern *et al*, 1999) that have reported equivalence in terms of both efficacy and safety for an in-patient combination oral antibiotic regimen as compared with standard in-patient parenteral regimen. However, the general applicability of oral therapy has been challenged in view of the relatively high levels of gastrointestinal toxicity of the oral regimen and the high rates of use of colony-stimulating factors (CSFs) in these study populations.

Oral antibiotic therapy has been evaluated in the outpatient setting by a number of groups but with variable findings. Malik *et al* (1995) compared outpatient oral antibiotic treatment with in-patient oral antibiotic treatment in an unselected group of cancer patients, including patients with acute leukaemia. Efficacy in the two groups was similar; however, 21% of the patients allocated to the outpatient group required hospitalisation and the study reported a 4% mortality rate, raising some concerns about the safety of this approach.

Serial studies at the MD Anderson Center (Rubenstein *et al*, 1993; Elting *et al*, 1997) using both intravenous and oral regimens in the outpatient setting have reported both high response rates and low readmission rates. However, these studies have not evaluated outpatient therapy in a randomised comparison with standard in-patient intravenous treatment.

Based on our own experience of managing low-risk neutropenic fever (Marshall *et al*, 2000), we initiated a single-centre randomised trial comparing a combination oral antibiotic regimen of ciprofloxacin and amoxicillin-clavulanate with early hospital discharge *vs* conventional in-patient management using a standard combination of intravenous antibiotics. The primary objectives of this study were to assess the efficacy and safety of this approach. Secondary objectives included duration of hospitalisation, readmission rates, patient acceptability and resource utilisation.

## PATIENTS AND METHODS

### Patient eligibility

Patients were recruited from Clatterbridge Centre for Oncology, a regional cancer facility treating solid tumours and lymphomas serving a population of 2 million people in Merseyside, UK. Patients undergoing conventional dose cytotoxic chemotherapy (i.e., nonmyeloablative chemotherapy not requiring routine use of growth factors given according to the internationally accepted protocols) were recruited between February 1997 and September 2000.

Neutropenia was defined according to standard criteria (Hughes *et al*, 1990, 1997) as an absolute neutrophil count (ANC) ⩽0.5 × 10^9^ l^−1^, but patients were also eligible if their ANC was ⩽1 × 109 l^−1^, but anticipated to fall to ⩽0.5 × 10^9^ l^−1^ within 24 h of entry into the study. Fever was defined as a temperature of ⩾38^o^C on two oral measurements 4 h apart within a 24 h period, one of which could have been measured by the patient prior to admission, or ⩾38.5^o^C on one occasion. It was also required that patients should have an anticipated duration of neutropenia of no longer than 7 days. Patients could be entered more than once following subsequent episodes of febrile neutropenia.

Patients were required to be haemodynamically stable with no signs or symptoms that required intravenous fluid support. Adequate renal function and the ability to maintain satisfactory oral intake were therefore required.

Patients were not eligible if they had undergone autologous bone marrow or peripheral blood stem-cell transplantation or had received antibacterial medication within 7 days of enrolment. The use of CSFs and cytokines was not permitted.

Further exclusion criteria included (i) any coexisting medical condition that would require in-patient treatment or monitoring, (ii) clinically documented infection, in the opinion of the investigator, likely to require targeted or prolonged duration of antibiotic therapy (e.g. cellulitis, abscess, pneumonia, CVC tunnel infection), (iii) inability to tolerate oral medication, and (iv) known allergy to study drugs.

Finally, all patients were required to have a responsible adult living with them who would be prepared to act as a carer if the patient were eligible for early discharge. Either patient or carer was also required to be able to read a thermometre. Patients with a history of poor compliance were excluded. All patients were 18 or over and gave written informed consent.

### Randomisation

All patients were initially assessed with a history and full physical examination. Standard screening investigations consisted of a full blood count and differential, a biochemical screen and a minimum of one set of peripheral venous blood cultures in addition to cultures via a central venous catheter if present. Chest radiographs and other microbiological cultures were performed only where clinically indicated.

Eligible patients were randomly assigned by means of consecutively drawn sealed envelopes to receive either an oral regimen of ciprofloxacin 750 mg every 12 h plus amoxicillin–clavulanate (amoxicillin 500 mg+clavulanate 175 mg) every 8 h for a total of 5 days, or an intravenous regimen of gentamicin 80 mg every 8 h and dose adjusted according to therapeutic levels plus tazocin (piperacillin 4 g+tazobactam 500 mg; Lederle, Maidenhead, UK) every 8 h until hospital discharge.

### Assessment and monitoring

Patients randomised to the intravenous arm were subject to a standard protocol for the management of neutopenic fever, with daily clinical assessment, repeat full blood count and differential at 48 h intervals, repeat blood cultures for patients with persistent fever and any additional investigations as clinically indicated. Patients were eligible for discharge when afebrile for 24 h with a rising neutrophil count (irrespective of the absolute value). Patients did not routinely receive antibiotics on discharge. Indications for changes in the treatment regimen included persistent fever ⩾72 h, positive culture results with resistant organisms, or clinical deterioration at the discretion of the treating clinician.

Patients randomised to the oral arm were eligible for discharge following 24 h of hospitalisation, if clinically stable and symptomatically improved, and according to the patient's wishes. On discharge, patients were supplied with a daily diary to record their temperature at 6-hourly intervals and any associated symptoms, and telephone contact was maintained with a member of the clinical research team. They were also given oral and written instructions with a 24-h contact telephone number at the specialist centre, emphasising the need for early reporting of any symptomatic deterioration. After discharge, patients were reviewed 7–10 days later in the oncology outpatient department to ensure full haematological recovery, assess outcome and determine further oncological management.

Those patients randomised into the oral arm who were not discharged after the 24 h assessment were reassessed daily including their eligibility for discharge as described above. Criteria for alterations to the antibiotic regimen were as outlined in the intravenous arm together with the inability to tolerate oral medication for any reason. Patients in the oral arm who required readmission following early discharge had their antibiotic regimen altered at the discretion of the treating physician depending on the indication for readmission.

### Definition and assessment of study end points

The primary end points of the study were success and safety. ‘Success’ was defined according to EORTC guidelines (Cometta *et al*, 1995) as lysis of fever and resolution of symptoms and signs with no modifications to the initial antibiotic regimen and with no recurrence within 7 days. Safety was assessed by the frequency of serious medical complications and deaths.

Secondary end points were total duration of hospital admission, frequency of readmission, toxicity of treatment and resource utilisation. Toxicity of treatment was assessed according to the Common Toxicity Criteria of the National Cancer Institute. Patients in the oral arm who had been discharged before completion of the antibiotic regimen were given symptoms diaries for self-completion.

Resource utilisation was assessed by comparing the financial costs of neutropenic episodes and the nursing care required for episodes of neutropenic fever in both arms. Estimated hospitalisation costs were calculated using a mean cost per routine in-patient day as determined by previous studies (Leese, 1993) (including medical, nursing, paramedical services and supplies, and general services such as catering and laundering, but excluding pharmacy and pathology costs to exclude double counting) multiplied by the number of in-patient days (including days after readmission where appropriate). Antibiotic costs were calculated according to standard NHS charges. Costs incurred by diagnostic tests and other therapeutic interventions were assumed to be equivalent in both arms and hence were excluded from the calculations.

A comparison of nursing resources was made using ‘GRASP’ (GRASP Systems Nurse Consulting International, CO, USA), an international nursing workload management system that is increasingly used within the NHS, to obtain an estimate of the nursing time required in ‘actual patient contact’.

### Statistics

From our own previous experience and previously published studies, and assuming a response rate of approximately 80% for the intravenous arm, this study was designed to enrol 63 episodes per arm to ensure that the oral arm would not be 20% worse (i.e. 60%, equivalent) at a level of significance *α*=0.05 with power of 80% using a two-sided *χ*^2^ test (N Query sample size program). In addition, first episodes of neutropenic fever (i.e. occurring in patients not previously randomised) were analysed separately to exclude any bias induced by repeat randomisation. We used the *χ*^2^ test or Fisher's exact test to compare various base-line characteristics and outcomes between the groups. The Mann–Whitney *U*-test was used to compare the continuous variables of neutrophil counts and stay length.

## RESULTS

### Patient characteristics

Between February 1997 and August 2000 111 patients, representing 135 episodes of fever associated with neutropenia, consented to participate in the study. Of the 135 episodes evaluated, nine episodes were excluded from the analysis, seven in the intravenous arm and two in the oral arm. Eight failed to meet the inclusion criteria; four were not neutropenic, one did not have fever, one required intravenous fluids at the time of randomisation, one had received antibiotics within 7 days of being entered into the study and one was allergic to study drugs. The final patient withdrew consent prior to commencement of antibiotics. The remaining 126 eligible episodes occurred in 102 patients (87 patients randomised once, 10 twice, three three times, one four times and one six times).

Sixty eligible episodes of neutropenic fever were assigned to the intravenous regimen of gentamicin and tazocin and 66 were assigned to receive the oral regimen of ciprofloxacin and amoxicillin–clavulanate. Of the 102 first episodes of neutropenic fever (i.e. occurring in patients not previously randomised), 51 were in the oral and 51 in the intravenous arm. The arms were well balanced with respect to age, sex and primary site of cancer, Table 1

**Table 1:** Patient characteristics

	**Intravenous arm (tazocin+gentamicin, *n*=60)**	**Oral arm (ciprofloxacin+amoxicillin–clavulanate, *n*=66)**
Age (years)		
Median (range)	50 (18–76)	53 (18–78)

Sex		
Female, no. (%)	37 (61.7)	41 (62.1)

Diagnosis, no. (%)		
Small-cell lung cancer	26 (43.3)	27 (40.9)
Breast	14 (23.3)	22 (33.3)
Testicular	2 (3.3)	3 (4.5)
Lymphoma	2 (3.3)	4 (6.1)
Other	16 (26.7)	10 (15.2)

Neutrophil count at presentation		
Median (range)	0.15 (0–0.9)	0.1 (0–0.8)

Neutrophil count ⩽0.5 × 10^9^ l^−1^		
No. (%)	55 (91.7)	61 (92.4)

Symptoms at presentation		
Mild–moderate, no. (%)		
Mucositis	28 (46.7)	30 (45.5)
Gastrointestinal	4 (6.7)	7 (10.6)
Respiratory	15 (25)	21 (31.8)
Genitourinary	6 (10)	4 (6.1)
No symptoms	23 (38.3)	23 (34.8)

a There were no significant differences between the two groups.

.

The majority of episodes occurred in women, reflecting the most frequent underlying diagnoses of breast cancer and small-cell lung cancer. Only 4.8% of episodes had a diagnosis of lymphoma, 92% of episodes had neutrophil counts of ⩽0.5 × 10^9^ l^−1^ at randomisation.

Clinical symptoms at randomisation were by definition mild to moderate (CTC grades I–II), Table 1. A total of 36.5% had no symptoms other than fever. Positive microbiological cultures are shown in Table 2

**Table 2:** Episodes with positive microbiological cultures

**Study arm**	**Source**	**Organism**	**Outcome**	**Further detail**
Intravenous	Peripheral blood culture	*Pseudomonas aeruginosa*	Success	
Intravenous	Peripheral blood culture	Coagulase negative staphylococcus^a^	Success	
Oral	Peripheral blood culture	Anaerobic diphtheroid^b^	Success	
Intravenous	Stool	*Giardia lamblia*	Failure	Patient developed diarrhoea after admission. Recent travel overseas. Resolution with oral metronidazole
Intravenous	Wound swab	*Staphylococcus aureus*	Success	
Intravenous	Urine	Faecal streptococcus	Failure	Persistent fever despite sensitivity of organism to study antibiotics. Resolved with addition of ceftazidime.
Intravenous	Stool	*Clostridium difficile* toxin	Failure	Patient developed diarrhoea after admission, nosocomial infection. Resolution with oral metronidazole.
Intravenous	Sputum	*Moraxella catarrhalis*	Success	

a One positive culture.

b One positive culture, non-JK group.

. Eight patients had positive microbiological cultures, of which two were isolated from a single culture and therefore of questionable significance. Five had documented clinical infections with positive cultures and the final patient developed *Clostridium difficile* associated diarrhoea following hospitalisation (superinfection).

### Efficacy and safety

The success rate to initial antibiotic therapy was similar in both groups. Treatment was successful in 90% of all episodes of neutropenic fever in the intravenous arm (95% confidence intervals 82.4–97.6%) and 84.8% of all episodes of neutropenic fever in the oral arm, *P*=0.55: absolute difference between the groups is 5.2%; 95% confidence interval for the difference −7 to 17.3%. Treatment was successful in all episodes with positive blood cultures. The success rates in the 102 first episodes of neutropenic fever were very similar: 45 of the 51 (88.2%) first episodes of neutropenic fever in the intravenous arm were successful, while 43 of the 51 (84.3%) first episodes in the oral arm were successful, *P*=0.77.

Episodes of neutropenic fever deemed failures are summarised in Table 3

**Table 3:** Reasons for failure of initial antibiotic regimen

	**Intravenous arm**	**Oral arm**
Death	1	0
Serious complication or clinical deterioration		
While in-patient	0	1
While outpatient	0	0

Intolerance of antibiotics due to		
Vomiting	0	1
Development of severe oesophagitis	0	2

Persistence of fever		
With microbiological evidence of resistance	2	0
Without microbiological evidence of resistance	3	6

. In the intravenous arm there was one death. This patient was undergoing palliative treatment with single agent doxorubicin for metastatic cystosarcoma phylloides and on admission had no symptoms of infection other than fever and no positive bacterial cultures. Both her fever and neutropenia had resolved prior to discharge. This patient died on the day following hospital discharge, 3 days following randomisation. The death was unexpected and was thought to be a consequence of a pulmonary embolus; however, post-mortem examination was not carried out. There were no other serious medical complications in the intravenous arm, and in all other patients there was complete resolution of the episode of febrile neutropenia without sequelae. Five episodes required changes to the antibiotic regimen because of persistent fever, Table 3.

In the oral arm there were no deaths. A total of 10 episodes of neutropenic fever in the oral arm required modification of the initial antibiotic regimen and were therefore deemed failures, Table 3. In only one of these, there was a serious medical complication: this patient developed hypotension within 12 h of admission. He was given intravenous fluid support and converted to intravenous antibiotics. Subsequently he developed the symptoms and signs of classical lobar pneumonia and required prolonged in-patient treatment; he was eventually discharged at 16 days after randomisation.

Of the remaining nine failures in the oral arm, all were converted to intravenous antibiotic regimens. Indications for the modification to the initial antibiotic regimen were: intolerance of the oral regimen due to vomiting (one); development of severe oesophagitis (two) and persistent fever at 72 h without serious clinical deterioration (six).

Five patients in the oral arm required readmission to hospital. Four of these were deemed failures and are described above. The fifth patient was readmitted with chest pain diagnosed as a pulmonary embolism.

### Toxicity

Both arms of the study were very well tolerated. In the oral arm there was one episode (0.8%) of severe toxicity, CTC grade 3, in which a patient was unable to tolerate oral ciprofloxacin due to vomiting, thus requiring a change to intravenous antibiotics. Other toxicity in this arm was mild–moderate gastrointestinal toxicity that did not require a change in antibiotic regimen, 14 patients (21.2%) having CTC grade 1–2 diarrhoea and five patients (7.6%) having CTC grade 1–2 nausea/vomiting. In the intravenous arm, there were no episodes of toxicity of CTC grade>1.

### Duration of hospital stay

The median in-patient stay was 4 days in the intravenous arm (range 2–8) and 2 days in the oral arm (range 1–16 days), *P*<0.0005, Table 4

**Table 4:** Duration of hospital stay

	**Intravenous arm *n*=60**	**Oral arm *n*=66**
No. (%) of episodes with in-patient stay length^a^		
		
1 day	0	23 (34.8)
2 days	8 (13.3)	13 (19.7)
3 days	11 (18.3)	13 (19.7)
4 days	14 (23.3)	5 (7.6)
5 days	10 (16.7)	6 (9.1)
6 days	10 (16.7)	1 (1.5)
7 or more days	7 (11.7)	5 (7.6)

Median (range)	4 (2–8)*	2 (1–16)
Total number of in-patient days	265	199

* *P*<0.0005, Mann–Whitney *U*-test.

a Includes duration of initial admission and after readmission where appropriate.

. Overall, the oral antibiotic policy resulted in a reduction of 66 in-patient days (199 compared to 265).

### Resource utilisation

The financial costs of hospitalisation and antibiotic costs, together with the estimated direct patient contact nursing hours required in the two arms are given in Table 5

**Table 5:** Resource utilisation

	**Intravenous arm *n*=60**	**Oral arm *n*=66**
Approximate costs of febrile neutropenic episodes (£)		
Hospitalisation	38 690	29 050
Antibiotics	11 690	2 060
Total	50 380	31 110
Mean, per episode	840	470

Approximate number of nursing hours ‘in direct patient contact’		
Total	1258	733
Mean, per episode	21	11

. Overall the costs of hospitalisation and antibiotics was more than £19 000 less in the oral arm compared with the intravenous arm, each episode in the oral arm costing approximately 56% of an episode in the standard management arm. Similarly, the estimated number of ‘direct patient care hours’ per episode in the oral arm was less than half of each episode in the intravenous arm (11 compared with 21).

## DISCUSSION

The treatment of neutropenic fever has improved considerably since its importance as a cause of morbidity and mortality in cancer patients undergoing chemotherapy was first highlighted more than 30 years ago (Bodey, 1966). Overall, fewer than 10% of patients with febrile neutropenia now die as a result of their infection and it is now possible to identify a subgroup of patients who are at particularly low risk of developing serious complications or death (Klastersky *et al*, 2000). This has in turn led to the development of less-intensive treatment strategies for low-risk patients including changes in antimicrobial therapy (combination *vs* monotherapy), changes in route of administration (intravenous *vs* oral) and treatment setting (in-patient *vs* outpatient). Response rates and safety have remained the main end points for antibiotic research in this area. However, as a consequence of the high response rates of newer antimicrobials and the low incidence of complications in this population, the safety and efficacy of different regimens are very similar (Elting and Cantor, 2002). Hence, in a manner analogous to the development of cytotoxic chemotherapy regimens, additional outcome measures need to be considered to differentiate between treatment strategies. These may include factors such as time to antibiotic response, total duration of hospitalisation, readmission rates, toxicity, convenience, resource utilisation and impact on quality of life.

Here, we present the first study to our knowledge comparing conventional in-patient treatment with standard intravenous antibiotics with oral antibiotics in conjunction with early hospital discharge in patients with low-risk neutropenic fever.

Previous research in this area has been hampered by the lack of an agreed definition of ‘low-risk’. This has in part been overcome by the recent introduction of a ‘risk index’ scoring system by the multinational association for supportive care in cancer (MASCC), (Klastersky *et al*, 2000), shown inTable 6

**Table 6:** Multinational association for supportive care in cancer scoring system for the proposed risk index for identifying low-risk febrile neutropenic patients (Klastersky *et al*, 2000)

**Characteristic**	**Score**
Burden of illness: no or mild symptoms^a^	5
No hypotension	5
No chronic obstructive pulmonary disease	4
Solid tumour or no previous fungal infection	4
No dehydration	3
Burden of illness: moderate symptoms^a^	3
Outpatient status	3
Age < 60 years	2

a Points attributable to burden of illness are not cumulative. The maximum theoretical score is therefore 26. The authors used a threshold of ⩾21 points to define ‘low-risk’.

. This study preceded the publication of this risk index, and therefore we have retrospectively scored our patients' baseline characteristics at randomisation. While we acknowledge that such retrospective analysis has limitations, we have found that more than 95% of patients in this study had scores ⩾21, a threshold which was suggested by the authors for defining patients at low risk of complications.

The definition of ‘low-risk’ used in the present study was based on the original definition proposed by Talcott *et al* (1988). However, given our intention of early hospital discharge and the high incidence of complications and readmissions in Talcott's initial pilot study of outpatient treatment (Talcott *et al*, 1994), we felt that careful patient selection was essential. We therefore extended the definition to exclude central venous catheter infections, pneumonia and cellulitis for which the choice of antibiotic and proposed short duration of antibiotic therapy may not be appropriate. We also included the previously recognised criterion of an expected duration of neutropenia of <7 days (Rubin *et al*, 1988). Although the anticipated duration of neutropenia was not found to be predictive of the likelihood of serious medical complication in the MASCC risk index, it was predictive of a higher probability of response to empirical antibiotic therapy without the need for modification, justifying our inclusion of this factor in our study.

Another important factor in patient selection in this study is that no patients received haematopoietic CSFs. This is in accordance with the ASCO 2000 guidelines (Ozer *et al*, 2000) which recommend that they should not be routinely used as adjunct therapy for the treatment of uncomplicated fever and neutropenia. This is in contrast to the two large randomised studies of oral *vs* intravenous antibiotics (Freifeld *et al*, 1999; Kern *et al*, 1999) in which CSFs were administered in 63 and 86% of episodes, respectively.

Inevitably as a consequence of the strict criteria used to define ‘low-risk’ in this study, the incidence of positive blood and other cultures was low and the large majority of patients did not have a defined site of infection. Nevertheless, the appropriate treatment for this group of patients remains an important clinical question since in most series of febrile neutropenia around two-thirds of patient episodes fit into this category.

The success rate without modification of the initial antibiotic regimen of the oral arm of this study was similar to the intravenous arm, 84.8 and 90%, respectively. These success rates are consistent with those of the largest open-label study (Rubenstein *et al*, 1993; Talcott *et al*, 1994; Kern *et al*, 1999), which has demonstrated equivalence of oral antibiotics compared with intravenous treatment in the in-patient setting. In our study, there was some evidence that clinicians were more confident of the intravenous regimen than the oral regimen. Firstly, there was an excess of failures in the oral arm due solely to persistent fever in the absence of clinical deterioration or resistance (Table 3). In addition, there was a greater number of patients in the intravenous arm who remained as in-patients beyond 4 days than one might have anticipated, given the 90% success rate of this arm. The majority of these patients had not been discharged because they did not fulfil the criteria for discharge of a rising neutrophil count. However, we identified five episodes in which patients had remained pyrexial for more than 72 h without antibiotic modification due to clinical well being. There were no such episodes in the oral arm. Hence, the success rate of the intravenous arm may have been somewhat overestimated.

More importantly, we encountered no significant problems with safety of the oral antibiotics and early discharge strategy: only one patient receiving oral antibiotics had significant clinical deterioration and this occurred within the initial 24-h mandatory monitoring period. However, we acknowledge that given the relatively small size of this study, there was limited power to detect small but clinically important differences in safety. In addition, in our study, all patients fulfilled strict entry criteria including good history of compliance and supportive home environment with continued contact by telephone with the specialist centre after discharge and rapid access for readmission to the centre in the event of deterioration. In view of this, our follow-up programme was less stringent than recently published guidelines suggest (Feld *et al*, 2002). Although there are presently no evidence-based criteria for optimal monitoring of patients postdischarge, we believe that more robust follow-up guidelines should be considered in the setting of less-specialised centres or multicentre trials.

Perhaps a more useful outcome measure than the conventional response to the initial antibiotic regimen is the success of the early discharge policy, as judged by the low readmission rate. Only five episodes (7.6%) required readmission, one of these for reasons apparently unrelated to the neutropenic episode. We used the criteria of clinical stability as judged by individual clinicians together with patients' symptomatic response to determine whether patients were eligible for early hospital discharge. This use of patients' subjective response is supported by a study from the MD Anderson Cancer Center (Elting *et al*, 2000). In this study, data from episodes of febrile neutropenia included in previous trials were examined to determine the value of various clinical criteria in predicting the outcome of the episode, that is, whether the episode resolved without antibiotic modification. The authors found that adding subjective patient response to objective measures of fever lysis substantially improved specificity of prediction of overall success of an antibiotic regimen with little loss of sensitivity.

The majority of patients admitted with complications of chemotherapy are receiving palliative treatment. Thus, the shorter duration of hospitalisation in the oral arm may be anticipated to impact upon patients' quality of life. Unfortunately, currently available instruments designed for measuring quality of life (Zigmond and Snaith, 1983; de Haes *et al*, 1990; Aaronson *et al*, 1993; Cella *et al*, 1993) are designed for comparison over weeks or months. Therefore, measurements in febrile neutropenia, where onset of infection and resolution of an episode may be only a few days apart, are unlikely to be helpful. Accurate assessment of the potential impact on patients' quality of life of this approach to the management of febrile neutropenia will require the development of more sensitive instruments. However, patient acceptability is perhaps a surrogate marker of quality of life. Thus by enabling clinically eligible patients to be involved in discharge planning they are able to make their own judgements about whether early discharge or continued in-patient treatment would be preferable. Of note is that while many patients expressed their preference for early discharge, a minority chose to remain in hospital for additional social and psychological support until the episode had completely resolved. Hence, early discharge is not a preferred option for all patients.

Toxicity of treatment is also an important end point and is also likely to impact upon patients' quality of life. In this study, toxicity in the oral arm was generally mild and consisted of easily managed nausea and diarrhoea. Only one patient required antibiotic modification because of toxicity. Toxicity was less than was reported in the two largest studies comparing oral and intravenous regimens (Freifeld *et al*, 1999; Kern *et al*, 1999), a discrepancy presumably partly attributable to under-reporting of adverse events by patients after discharge.

Resource implications of different therapeutic strategies are increasingly important in all healthcare systems. The major financial burden of conventional treatment of neutropenic fever is the cost of in-patient care, estimated to be between 58 and 78% of the total cost. (Leese, 1993; Rubenstein *et al*, 1993; Dranitsaris *et al*, 1995). In this study, the duration of hospital stay in the oral arm was significantly shorter than in the intravenous arm with a median in-patient stay of 2 days compared with 4 days. Overall, this gave a saving of 66 days of hospital stay in the oral compared with the intravenous arm. This is reflected in the comparison of the costs of the two arms of the study. It should be noted that the stay length in the intravenous arm is already shorter than previous studies, which estimated a mean stay of 6.3 days (Leese, 1993). This is because our ‘standard’ management guidelines already incorporate a policy of discharge when patients are afebrile for 24 h and with a rising neutrophil count, irrespective of the absolute value (Marshall *et al*, 2000). While this approach appears less stringent than published guidelines (Hughes *et al*, 2002), it represents a policy derived at a specialist centre with considerable experience in the management of low-risk patients. Hence, the savings in hospital admission days using oral antibiotics with early discharge would be greater if compared with more conventional management approaches. In addition, cost per in-patient day varies greatly, both between and within countries. Therefore, the potential savings of introducing this treatment approach would be dependent on existing local policies and circumstances.

The introduction of oral antibiotics and early hospital discharge may have additional, though less easily quantifiable, benefits with respect to nursing and pharmacy time. Oral therapy clearly negates the requirement for care of intravenous cannulae as well as aseptic reconstitution. Assessment using the GRASP nursing management tool suggested that this treatment approach might reduce number of nursing hours required in ‘direct patient care’ by more than half.

In conclusion, our study suggests that oral antibiotics in conjunction with early hospital discharge for patients who remain stable after a 24 h period of in-patient monitoring offers a feasible and cost-effective alternative to conventional management of low-risk neutropenic fever. However, we urge caution when applying these findings outside the setting of a single specialist centre. We also recognise that the power of this study to detect small but clinically important differences in safety is limited. We therefore believe that the results of this study should provide a platform for larger trials to further evaluate the policy of oral antibiotics with early discharge in the multicentre setting.
